# The Paradox of High Availability and Low Recognition of Soluble HLA-G by LILRB1 Receptor in Rheumatoid Arthritis Patients

**DOI:** 10.1371/journal.pone.0123838

**Published:** 2015-04-08

**Authors:** Tiago Degani Veit, José Artur Bogo Chies, Magdalena Switala, Bettina Wagner, Peter A. Horn, Mauricio Busatto, Claiton Viegas Brenol, João Carlos Tavares Brenol, Ricardo Machado Xavier, Vera Rebmann

**Affiliations:** 1 Laboratório de Imunogenética, Universidade Federal do Rio Grande do Sul, Porto Alegre, Brazil; 2 Institute for Transfusion Medicine, University Hospital of Essen, Essen, Germany; 3 Serviço de Reumatologia, Hospital de Clínicas de Porto Alegre, Porto Alegre, Brazil; University of London, St George's, UNITED KINGDOM

## Abstract

HLA-G is a regulatory molecule involved in immunologic tolerance. Growing evidence indicates that HLA-G plays a role in the regulation of inflammatory processes and autoimmune diseases. This study aimed at a systematic evaluation of soluble HLA-G (sHLA-G) in plasma of rheumatoid arthritis (RA) patients with long-lasting chronic inflammation. RA patients (n=68) and healthy controls (n=26) had their plasmatic sHLA-G measured by ELISA whereas the binding capability of sHLA-G to its cognate LILRB1 receptor was measured by a Luminex-based assay. All subjects were PCR-genotyped for *HLA-G* 14bp polymorphism (rs66554220). Significantly higher sHLA-G levels were observed in patients (p<0.001), however no significant differences were observed in LILRB1 binding capacity between RA patients and controls. Remarkably, the proportion of patients presenting specific binding of sHLA-G to LILRB1 was significantly decreased as compared to controls (56% vs. 81%, p=0.027). Patients without rheumatoid factor (RF-) were significantly overrepresented in the group of patients positive for LILRB1 binding as compared to patients without LILRB1 binding (31% vs 10%, p=0.033). Furthermore, methotrexate treated patients (n=58) revealed significantly lower LILRB1 binding to sHLA-G molecules than non-treated patients (medians: 12.2 vs. 67.7 units/ml, p=0.031). Unlike in controls, no significant differences in sHLA-G levels were observed among patients grouped by 14pb genotype. Thus, in a substantial number of late RA patients, the circulating sHLA-G molecules are impaired regarding LILRB1 recognition, meaning that although increased levels are observed; these molecules are not qualified to exert their protective functions against inflammation. Our findings offer new insights into the immunopathology of RA patients with long-lasting anti-RA-treatment and highlight the importance to also measure the binding capability of sHLA-G to LILRB1.

## Introduction

HLA-G is a non-classical HLA class I molecule, which was first characterized by its expression at the maternal-fetal interface, limited tissue distribution in healthy conditions and by the expression of seven different isoforms [1–3] that can be either membrane-bound (G1–G4) or secreted (G5–G7). In principle, all membrane-anchored molecules can be released from the cell surface through shedding. The stability of HLA-G mRNA is associated to certain polymorphic variants in 3´UTR of the *HLA-G* gene. The homozygous deletion of 14 bp at that region (rs66554220) is described to confer a more stable mRNA as compared to the homozygous insertion genotype [4].

Since it was first described in cytotrophoblasts, this molecule has attracted much attention due to its immunotolerogenic properties. HLA-G and its soluble forms (sHLA-G) are capable of interacting with several receptors (LILRB1, LILRB2, KIR2DL4, CD8, CD160), which are present in a variety of cells of the immune system, such as NK cells, T and B lymphocytes and antigen-presenting cells (APCs) [5].

Several immunosuppressive mechanisms mediated by HLA-G/sHLA-G molecules were described to date: the inhibition of cytotoxicity, proliferation and/or differentiation of T cells, induction of tolerogenic APCs or suppressive T and NK cells, induction of apoptosis, as well as up regulation of inhibitory receptors [6–8]. All these features have made HLA-G a key molecule in situations where immune tolerance is needed, such as pregnancy and its complications, transplantation, cancer and viral infections. A unique feature of HLA-G/sHLA-G among other HLA molecules is that it is capable of dimerizing with itself, displaying a higher affinity for its cognate receptors [9]. Thus, the supply of sHLA-G dimers may regulate its immune suppressive potential [10, 11].

In recent years, a substantial number of scientific studies have indicated that the expression of HLA-G plays a role in the regulation of inflammation in autoimmune diseases [7, 12, 13]. The first studies in this area described the HLA-G expression in muscle fibers in various inflammatory myopathies, in atopic dermatitis and psoriatic skin [14–16]. It was promptly proposed, based on the finding that HLA-G seems to shift T-helper responses towards a Th2-type response, that it would act as a tissue-protective molecule in inflammatory responses and numerous studies in this area have been performed since then [17–21].

Rheumatoid Arthritis (RA) is a chronic systemic inflammatory disease that can lead to joint deformities and permanent physical disability. The role of HLA-G in the pathology of RA has so far almost exclusively been investigated in the early phase of RA pointing to the fact that soluble HLA-G (sHLA-G) levels will be up-regulated by the patient’s response to disease modifying anti-rheumatic drug (DMARD) therapy [22–24]. All these observations are in agreement to the suggested role of sHLA-G as an immune tolerogenic molecule in the context of RA. Thus, sHLA-G levels and the 14 bp polymorphism are suggested as prognostic factors stratifying patients in groups of responders and non-responders to anti-RA therapy at the onset of the disease. In this context, a question that still lacks sufficient assessment is, how does HLA-G behave in RA patients after long-lasting disease course and DMARD treatment, i.e. could HLA-G be playing a relevant immune protective role in RA as the disease progresses? In order to address this, we performed a systematic evaluation of sHLA-G with regard to its circulating levels, its capability to bind its cognate receptor *leukocyte immunoglobulin-like receptor subfamily B member 1* (LILRB1—also known as ILT2, LIR1, MIR7, CD85d) and the genetic control of its release. We observed high sHLA-G levels in patients with continuous anti-RA-treatment but the sHLA-G molecules were not recognized by LILRB1 in a substantial number of patients suggesting that these molecules are not qualified to exert its immune suppressive and protective function against inflammation via LILRB1.

## Materials and Methods

### Subjects and plasma sampling

Plasma samples were obtained from 68 RA patients (58 women and 10 men) diagnosed according to the American College of Rheumatology’s criteria for the classification of Rheumatoid Arthritis (RA) [23]. Patients having another connective tissue disease, other than secondary Sjögren syndrome, unresolved malignancies or acute infections were excluded from the study. Patients were followed at the Rheumatology Outpatient Clinic of the Hospital de Clínicas de Porto Alegre. The disease activity scores involving 28 joint counts (DAS28) were assessed during the study period. At each visit, clinical assessment consisted of swollen and tender joint counts at 28 joints, pain visual analogue scale (VAS), evaluator and patient global assessment of disease activity by VAS, health assessment questionnaire (HAQ) [25], C-reactive protein (CRP) levels and erythrocyte sedimentation rate (ESR). Plasma samples from 26 healthy individuals (21 women and 5 men) served as controls.

Patients had their medical records reviewed for further clinical and radiographic data. Clinical data included atlantoaxial subluxation and extra-articular (EA) manifestations (rheumatoid nodules, amyloidosis, vasculitis, pneumonitis and episcleritis). Erosive disease was characterized by the presence of erosions in any of the hands and feet x-rays. The demographical profiles of the RA patients and adult controls are shown in Table 1. At the time of sampling, 58 patients were taking methotrexate, 39 were under treatment with prednisone, 29 with NSAID (non-steroidal anti-inflammatory drugs), 15 with leflunomide, 6 with anti-malaric drugs (chloroquine diphosphate or hydroxyhloroquine), 4 with sulphasalazine, and 3 patients were on biological therapy with infliximab. The study was approved by the Ethics Committee of Hospital de Clínicas de Porto Alegre (Project 08–366). All subjects gave their written informed consent to participate in this study and all experiments were performed in compliance with the Helsinki Declaration (http://www.wma.net/en/30publications/10policies/b3/index.html).

**Table 1:** Demographic profile of RA patients and healthy controls.

	RA (N = 68)	HC (N = 26)
**Male: Female**	10: 58	5: 21
**European-derived (%)**	63 (91.3)	26 (100)
**Age±SD (years)**	57.4±10.6	48±15.2
**Disease duration (years)**	12.0±9.4	
**DAS28 ESR(n)**	4.05±1.55 (59)	
**DAS28 CRP(n)**	3.64±1.28 (57)	
**CDAI (n)**	15.21±10.54 (58)	
**HAQ (n)**	1.18±0.76 (56)	
**Rheumatoid factor positivity (%)**	56 (78.9)	
**Bone erosions (%)**	61 (87.1)	
**Rheumatoid nodules (%)**	14 (19.7)	
**Amiloidosis (%)**	2 (2.8)	
**Episcleritis (%)**	3 (4.2)	
**Sub-luxation (%)**	11(15.5)	
**Sjögren’s syndrome (%)**	2 (2.8)	

DAS = Disease Activity Score, CDAI = Clinical Disease Activity Index

HAQ = Health Assessment Questionaire

HC = healthy controls

### Quantification of sHLA-G

EDTA plasma samples were obtained from peripheral blood. The determination of sHLA-G was performed as described previously [26]. For sHLA-G ELISA the specific capture reagent was the monoclonal antibody G233 (Exbio, Czech Republic). Bound molecules were detected by a polyclonal antiserum rabbit anti-human β_2_-microglobulin (B2M) (Dako, Hamburg, Germany) followed by Envision goat anti-rabbit horseradish peroxidase (Dako, Germany). Plasma samples were diluted 1:2 in PBS. Purified sHLA-G5 protein served as standard reagent [27] and 3,3′,5,5′-tetramethybenzidine as substrate solution. After stopping the enzyme reaction with 1 M H_2_SO_4_, the optical density was measured at 450 nm (Biotek Instruments, Winooski, VT). Determination of plasma sHLA-G levels was performed by four-parameter curve fitting. HLA-G5 was used as standard in a concentration ranging from 0.4375–112 ng/ml. PBS was used as a negative control. For the calculation of the ELISA detection limits, a standard curve starting from a concentration of 8 ng/ml was performed in equimolar dilution steps of 5 and 1 ng/ml, respectively. The results obtained were subjected to the software DINTEST (Institute für Rechts-und Verkehrsmedizin, Universitätsklinikum Heidelberg, Germany). According to this procedure, the detection limit of sHLA-G ELISA was 0.94 ng/ml.

### Quantification of sHLA-G recognition by the LILRB1 receptor

**Fig 1:**
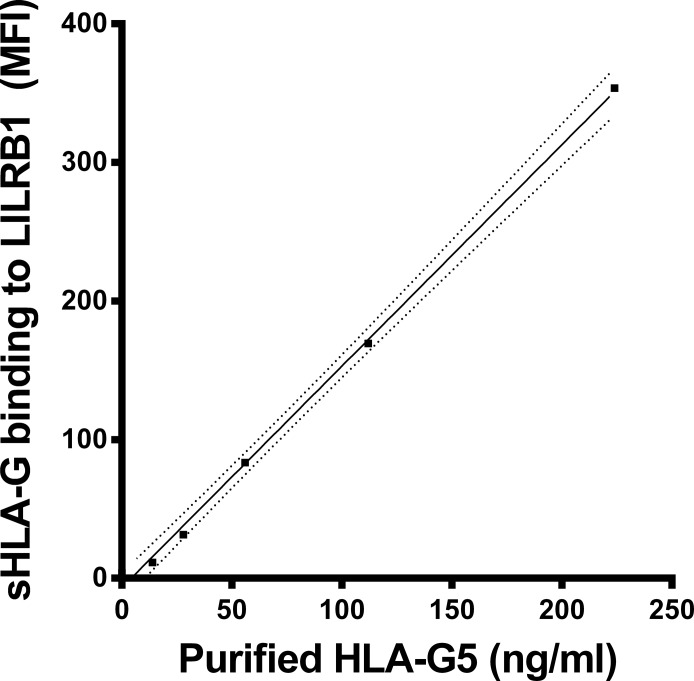
sHLA-G recognition by LILRB1 receptor. Straight line indicates the linear regression and dotted line indicates the 95% confidence interval of regression. MFI = mean fluorescence intensity. One unit/ml sHLA-G5 corresponds to 1ng/ml of purified sHLA-G5.

For the measurement of LILRB1 receptor recognition to sHLA-G molecules in plasma the Luminex-x-MAP technology and instruments were used (Luminex). Microspheres with color code 36 were covalently coupled with the G233 mAb [28]. Recognition of the mAb to the micropheres was performed as recently described [29]: G233 coupled microspheres (1250 per sample) were incubated in a total volume of 50μl with plasma diluted 1:4 in Luminex buffer (Cayman). Thereafter the bound HLA-G molecules were exposed to recombinant human LILRB1 receptor protein fused to the Fc region of human IgG1 (R&D Systems). Then, the bound LILRB1 receptor was detected by the anti-human LILRB1 mAb (BD Biosciences), PE conjugated. Measurement of the microspheres was carried out by the Luminex 100 IS System (Luminex). In total, the median fluorescence intensity from 100 microspheres was calculated in each sample. For the determination of HLA-G molecules recognized by the LILRB1 receptor, purified HLA-G5 was used in concentrations ranging from 0–224 ng/ml (Fig 1). The LILRB1 recognition of sHLA-G is given in fluorescence units (FU)/ml. One unit corresponds to 1 ng purified HLA-G5. The detection limit was 11.9 units/ml. Luminex buffer was used as a negative control.

### Polymerase chain reaction amplification of the 14bp polymorphism in exon 8 (3’UTR) of the *HLA-G* gene and genotyping

DNA was isolated from peripheral blood of patients and controls using a salting out method [30]. The genotyping of the 14bp polymorphism of the HLA-G gene was performed as previously described [31, 32].

### Statistical analysis

Statistical analysis was performed using SPSS 16.0 (SPSS Inc., Chicago, IL) or GraphPad Prism 6.0 (GraphPad Software, San Diego, CA). According to nonparametric distribution of sHLA-G plasma values, plasma levels of respective groups are presented as medians, and median differences between groups were assessed by using the Mann–Whitney U or Kruskall-Wallis H tests. Spearman’s nonparametric correlation coefficients and their p values have been calculated for sHLA-G and parameters of RA activity.

## Results

### Circulating sHLA-G levels are increased in late RA patients

**Fig 2:**
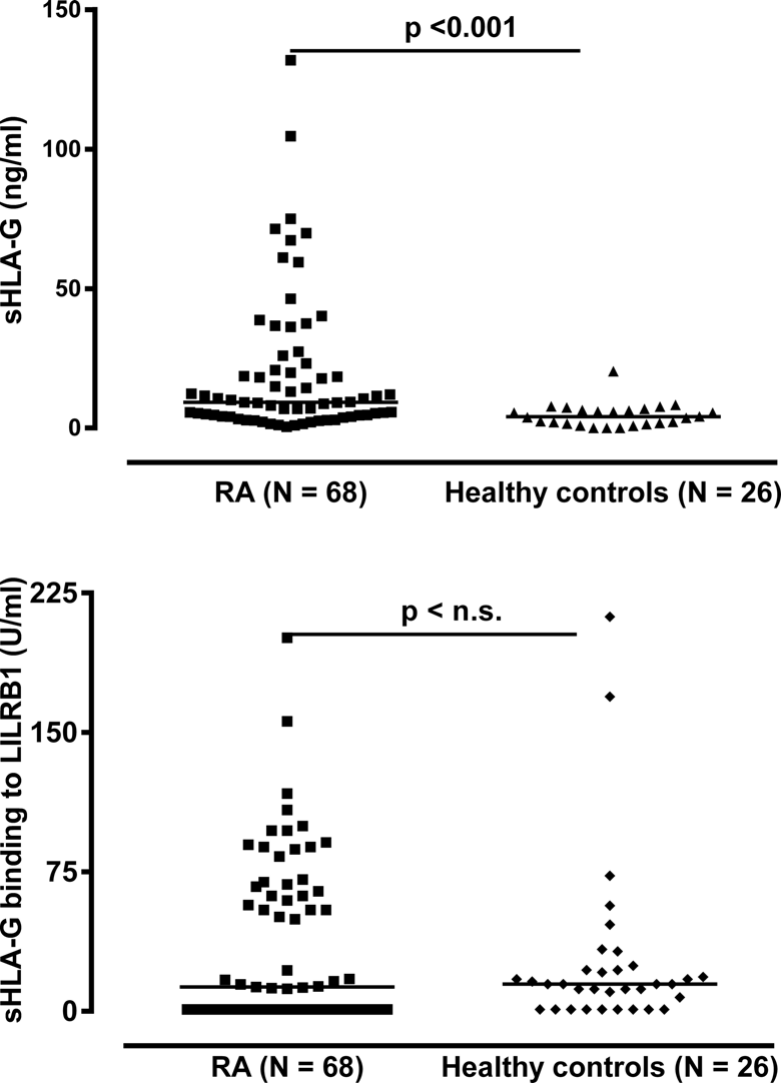
Soluble HLA-G level in plasma and its recognition by LILRB1 in RA patients and healthy controls. RA = Rheumatoid arthritis

In order to investigate whether sHLA-G levels were altered in the periphery among late rheumatoid arthritis patients, we analyzed sHLA-G in plasma samples in 68 RA patients with mean disease duration of about 12 years (Table 1). The sHLA-G plasma levels were significantly increased in RA patients as compared to healthy controls (p<0.001, Mann-Whitney test, Fig 2): The median sHLA-G level of RA patients was 9.3 ng/ml (range: 0–131.9 ng/ml) and in healthy individuals 4.6 ng/ml (range: 0–20.4 ng/ml). Of note, 18 patients (26.5%) exhibited sHLA-G levels above the controls’ highest concentration (Fig 2). With respect to sHLA-G levels and disease activity parameters, however, no significant correlations were observed (Table A in S1 File). In addition, no significant correlations could be observed between sHLA-G levels and anti-RA treatment regimen (Table B in S1 File).

### Circulating sHLA-G molecules are not recognized by their cognate LILRB1 receptor in a substantial number of late RA patients

**Fig 3:**
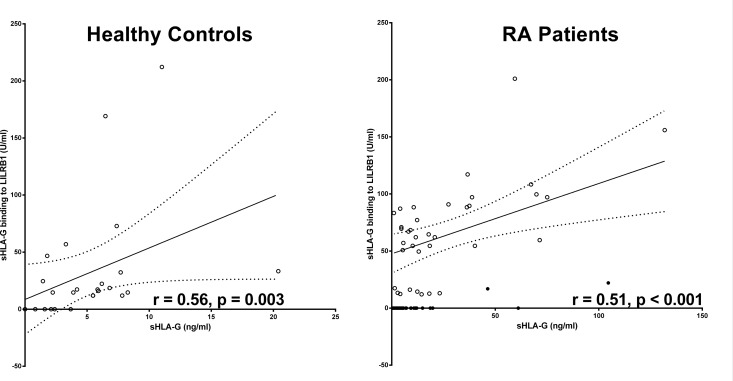
Correlation of sHLA-G levels and its recognition by LILRB1 receptor in RA patients and healthy controls. Straight line indicates the linear regression and dotted line indicates the 95% confidence interval of regression. Closed cycles indicate plasma samples of RA patients with HLA-G molecules with an impaired LILRB1 recognition.

In order to evaluate the potential recognition of sHLA-G molecules present in the RA patients plasma we analyzed the binding capacity of sHLA-G to its cognate LILRB1 receptor [28]. Importantly, in the recognition assay we used the same antibody (mAb G233) to capture sHLA-G as for the quantitative assessment, but this time bound molecules were exposed to recombinant human LILRB1 followed by anti-human LILRB1 antibody instead of polyclonal rabbit anti-human β_2_-microglobulin antiserum. Results of these assay showed that, despite the high sHLA-G levels in patients, no quantitative differences in recognition by LILRB1 were observed between patients and controls (Fig 2). The median in LILRB1 recognition was 12.9 FU/ml in the patients’ group and 15.3 FU/ml in the controls’ group (p = 0.632, Mann-Whitney test). However, the proportion of patients presenting specific recognition of sHLA-G molecules by LILRB1 (above the calculated detection limit of the test) was significantly decreased as compared to healthy controls (56% vs. 81%, p = 0.027, Chi-square test). Of note, among individuals with no detectable LILRB1 recognition, significantly higher median values of sHLA-G were identified in patients as compared to controls (5.42 vs. 0.83 FU/mL, p < 0.001, Mann-Whitney test, Table 2). This suggests that lack of LILRB1 recognition is mainly due to low amounts (not detectable) of sHLA-G in healthy individuals, whereas in RA patients it is rather a consequence of a large amount of non-recognized sHLA-G molecules. This assumption is further supported by the correlation analysis between sHLA-G levels and LILRB1 recognition: Although a significant positive correlation between sHLA-G level and LILRB1 recognition was found in both healthy controls (r = 0.57, p = 0.003) and RA patients (r = 0.52, p < 0.001), the correlation did differ with respect to the slope of their linear regression line (Fig 3): For healthy controls the slope of regression line was with 4.43 ± 2.2 steeply rising, whereas for RA patients the slope of regression line was with 0.96 ± 0.18, clearly decreased. In addition to 30 RA patients without detectable LILRB1 recognition, despite substantial levels of circulating sHLA-G molecules, two RA patients revealed sHLA-G molecules in a concentration above 45 ng/ml with a very weak recognition by the LILRB1 receptor. Thus, in a substantial number of late RA patients the circulating sHLA-G molecules in the blood were not or only hardly recognized by the LILRB1 receptor suggesting that these sHLA-G molecules are functionally inactive with regard to this receptor.

**Table 2:** Correlation of sHLA-G recognition by LILRB1 and sHLA-G levels.

LILRB1 recognition	N (%) RA	N (%) HC	sHLA-G (range) RA^a^	sHLA-G (range) HC^a^	P-value^b^
**Positive**	38 (56)	21 (81)	16.1 (1.1–131.9)	5.8 (1.4–131.9)	
**Negative**	30 (44)	5 (19)	5.4 (0.5–61.1)	0.8 (0–2.4)	**<0.001**
**P-value**	**0.032** ^**c**^				

^a^median (range) in ng/ml

^b^Comparison between patients and controls, Mann-Whitney test

^c^Comparison between patients and controls of LILRB1 recognition frequencies by Fisher’s exact test

HC = healthy controls

### RF- negative patients were significantly overrepresented in the group of patients positive for LILRB1 binding

LILRB1 recognition was also analyzed with respect to the presence and absence of rheumatoid factor (RF). The group of patients with detectable LILRB1 binding presented a higher proportion of RF-negative patients (31%) compared to the group of patients without LILRB1 recognition (10%, p = 0.033, Chi-square test, Table 3). Also, from the 5 patients where ACCP was accessed, four presented positivity to LILRB1 recognition and were simultaneously negative to ACCP, whereas the only patient negative to LILRB1 recognition was positive to ACCP.

**Table 3:** Correlation of sHLA-G recognition by LILRB1 and the presence of RF.

LILRB1 recognition	N (%) RF-positive	N (%) RF-negative	P-value^a^
**Positive**	27 (69)	12 (31)	**0.033**
**Negative**	27 (90)	3 (10)	

^a^Chi-square test

### Methotrexate treated patients presented lower LILRB1 binding to sHLA-G molecules

**Fig 4:**
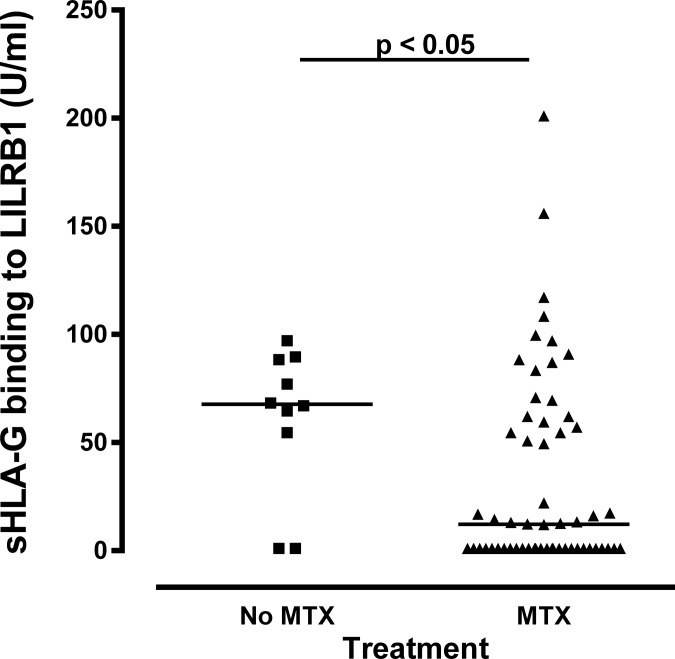
Methotrexate treatment and sHLA-G recognition by LILRB1 in RA patients.

As the anti-rheumatic drug methotrexate (MTX) is reported to mediate the up-regulation of interleukin-10 and HLA-G [33], we additionally analyzed the LILRB1 recognition to sHLA-G molecules in treated (N = 58) and non-treated (N = 10) RA patients. Interestingly, patients treated with MTX revealed significantly lower sHLA-G recognition of LILRB1 than non-treated patients (medians: 12.2 vs. 67.7 FU/ml, p = 0.040, Mann Whitney test, Fig 4).

### Circulating sHLA-G levels are not associated to genotypes of the 14bp polymorphism of the HLA-G gene

Given that polymorphisms at the *HLA-G* gene are potentially involved in the susceptibility to autoimmune diseases such as arthritis and were suggested as prognostic factors enabling to stratify patients in groups of responders and non-responders [23], HLA-G variants for the 14 bp were additionally taken into account in the present study (Table 4). No significant differences were observed in genotype frequencies between patients and controls. The already described difference in blood sHLA-G levels among 14 bp *HLA-G* genotypes was observed in healthy controls, with individuals homozygous for the insertion allele (ins/ins) presenting the lowest median values, as expected (0.7 vs. 5.9 in heterozygotes and 5.5 ng/mL in deletion homozygotes, p = 0.018). However, in late RA patients a different pattern of expression was observed: All three genotypes expressed comparable levels of sHLA-G (medians: 5.5, 10.6 and 6.4 ng/mL, respectively, p = 0.534 Mann Whitney test).

**Table 4:** HLA-G 14 bp genotype in relation to sHLA-G levels in adult patient groups and healthy controls.

			RA	HC		RA	HC	
HLA-G genotype	N (%) RA	N (%) HC	sHLA-G^a^ (range^b^)	sHLA-G^a^ (range^b^)	P_bonf_ ^c^	LILRB1 recognition^d^ (range^e^)	LILRB1 recognition^d^ (range^e^)	P_bonf_ ^c^
**del/del**	22 (32)	11 (42)	6.4 (1.5–131.9)	5.5 (1.6–11.0)	0.178	0 (0–155.9)	17.3 (0–212.2)	0.074
**del/ins**	37 (55)	11 (42)	10.6 (0.5–104.6)	5.9 (0.8–20.4)	**0.013**	16.1 (0–117.1)	14.7 (0–169.2)	0.970
**ins/ins**	9 (13)	4 (15)	5.5 (1.0–59.5)	0.7 (0–2.1)	**0.020**	50.8 (0–201.0)	3.8 (0–24.6)	0.148
**P-value**	0.567^f^		0.534^g^	**0.018** ^**g**^		0.200^g^	0.182^g^	

^a^median in ng/ml

^b^(minimum—maximum sHLA-G in ng/ml)

^c^Comparison between patients and controls of the same genotype, Mann Whitney test

^d^median in units/ml (measured by LUMINEX)

^e^(minimum—maximum fluorescence intensity)

^f^Comparison of genotype frequencies between patients and controls, Chi-square test

^g^Comparison among genotypes, Kruskall-Wallis test

HC = healthy controls

## Discussion

HLA-G has been described as a molecule involved in tissue protection against inflammatory aggression and its expression has been described in several inflammatory conditions, including multiple sclerosis, inflammatory bowel disease, RA and juvenile idiopathic arthritis [20, 22, 34, 35]. Despite of the fact that previous studies have already analyzed soluble HLA-G levels in RA plasma samples, this is the first study to investigate sHLA-G in terms of its binding capacity to one of its receptors (LILRB1) in this disease. From that analysis it was possible to identify an impaired binding capacity of sHLA-G circulating molecules to LILRB1 in RA patients, suggesting an impaired functionality of these molecules regarding this receptor.

The first important finding was that circulating sHLA-G levels were increased in late RA patients. This is in agreement with the results from Rizzo et al., which showed that in early untreated RA patients, detectable levels of sHLA-G in plasma could be observed in all subjects, as compared to a minority (23%) of healthy controls, and that those levels increased upon anti-RA treatment in those patients [23]. This increase could reflect an attempt of the immune system to counterbalance the autoimmune process. However, in our RA patient cohort, no noticeable correlation between plasma sHLA-G levels and disease activity parameters was observed (Table A in S1 File). Furthermore, in our study, sHLA-G levels did not differ with respect to anti-RA treatment. Our results are at variance to the first report on sHLA-G in RA describing lower plasma HLA-G levels in late RA patients [22] and to those from Rizzo et al. [23]. Nevertheless in the latter study only 23% of the controls were positive for sHLA-G whereas substantial amounts of sHLA-G molecules could be detected in all blood samples of RA patients (100%). The differences in mean sHLA-G levels might be partially explained by different protocols of the HLA-G measurement: while the study from Verbruggen et al. [22] used a two-step ELISA that included the depletion of classic HLA-I and HLA-E and detection by a pan-HLA-I antibody (W6/32), our assay and also that one used in the work from Rizzo et al. skipped the depletion step and used a more direct detection strategy by an anti-HLA-G antibody. Importantly, the study of Rizzo et al. [23] used the antibody MEM-G/09 as capture antibody where we were using the HLA-G specific antibody G233 and for these two antibodies discrepancies in sHLA-G concentration readouts have been previously reported [36]. Other explanations for those discrepancies might include sample composition and differences in treatment regimens.

The most intriguing observation from our study was that circulating sHLA-G molecules are not recognized by their cognate LILRB1 receptor in a substantial number of late RA patients. This observation was possible due to the application of a newly implemented Luminex assay [28] based on the use of microspheres being coated with the HLA-G specific antibody (G233 mAb), which has also been used for the quantitative determination of sHLA-G molecules by ELISA. However, the bound sHLA-G molecules were detected by a chimeric LILRB1 receptor. Thus, using this method it is possible to quantify specifically the presence of HLA-G molecules, which can be recognized by LILRB1. The obtained results emerge the question: Why sHLA-G levels do not correlate to LILRB1 recognition in certain RA patients? Dimer formation is a known feature of sHLA-G that greatly enhances its recognition to LILRB1 and LILRB2 receptors [9]. The conditions under which HLA-G molecules form dimers are not yet fully understood. It is known that dimerization occurs after passing through the Golgi apparatus [37], and in solid tumors it has been evidenced that HLA-G dimerization is enhanced by environmental factors such as interferon-β or- γ [38]. Moreover, superior long-term immunosuppressive effects of HLA-G dimers over monomers were already documented in a model of collagen-induced arthritis [39]. A higher abundance of HLA-G monomers or even other non-classical HLA-G-like structures [36] could explain why the sHLA-G levels observed in certain patients did not correlate with LILRB1 recognition. Of course it cannot be ruled out that the antibody G233 favors to bind to certain HLA-G structure e.g. monomers, dimers or HLA-G-like structures. However, this antibody was used as capture reagent in both assays, which should allow a correlation. Thus, a lack of correlation can only be attributed to differences in the binding of the detection reagents. Here, it cannot be excluded that G233 bound to HLA-G may block the LILRB1 binding site, preventing HLA G monomer binding by LILRB1 from being detected. Of note, the impaired LILRB1 recognition cannot be extended to LILRB2 and other HLA-G specific receptors without further examination. LILRB2, for instance, recognizes B2M-free forms of HLA-G, which could not be identified by our detection systems. However, these HLA-G structures might additionally play a functional role in the immune pathology of RA.

Our results also showed an overrepresentation of RF- patients in the group of patients with detectable binding of HLA-G to LILRB1. Recently, Naji et al. demonstrated that HLA-G recognition to LILRB1 suppresses B cell responses, including B cell proliferation, differentiation, and Ig secretion [40]. Therefore, we could suggest that in rheumatoid arthritis patients, high levels of sHLA-G molecules with capability to bind to LILRB1 could also impair the production of auto-antibodies. In this sense, and also considering the existence in the literature of controversial results in sHLA-G levels and severity in different autoimmune diseases (such as in Systemic Lupus Erythematosus [41, 42]), it would be important not only to access the presence and levels of sHLA-G in autoimmune patients but also to perform tests evaluating the its recognition by its cognate receptors.

Considering anti-RA therapy, patients treated with MTX revealed lower sHLA-G recognition of LILRB1. This suggests that MTX does not facilitate the up-regulation of HLA-G dimers, at least in late RA patients after long lasting treatment. Further studies need to be performed in order to clarify how HLA-G dimerization will behave in late RA patient treatment.

Previous studies have associated the 14 bp insertion allele and some *HLA-G* alleles linked to it as low sHLA-G producers, [4, 43, 44]. This finding was confirmed in our control sample but not among RA patients. This suggests that, despite being associated to lower levels of sHLA-G expression in plasma of healthy individuals, the 14 bp insertion allele (and consequently the ins/ins genotype) is responsive in situations in which sHLA-G mediated regulation of inflammation is required (i.e. immunologic stress). Furthermore, the lack of association between genotype and sHLA-G plasma levels in RA patients suggests that post-transcriptional mechanisms affecting both the level and function of sHLA-G might be operative in late RA.

## Conclusions

In this study we present for the first time that in a substantial number of chronically inflamed RA patients, the circulating sHLA-G molecules in the blood are impaired with respect to the LILRB1 receptor recognition. Thus, our findings offer new insights into the immune pathology of chronically inflamed RA patients after long-lasting anti-RA treatment. In this scenario, sHLA-G levels are indeed increased but these molecules are not qualified to exert its immune suppressive and protective functions against inflammation via LILRB1 receptor. Giving consideration to the inherent complexity of the HLA-G molecule, these observations call attention to the importance of receptor binding assays as a complementation to the quantification of HLA-G, in order for a better understanding the physiological phenomena involving this molecule.

## Supporting Information

S1 File Table A, Correlations of RA disease parameters and levels of sHLA-G molecules with their LILRB1 recognition. Table B, Relationship of RA treatment and levels of sHLA-G molecules with their LILRB1 recognition.
(DOCX)

## References

[1] Paul P, Cabestre FA, Ibrahim EC, Lefebvre S, Khalil-Daher I, Vazeux G, et al Identification of HLA-G7 as a new splice variant of the HLA-G mRNA and expression of soluble HLA-G5, -G6, and -G7 transcripts in human transfected cells. Hum Immunol. 2000;61: 1138–49. 11137219 10.1016/s0198-8859(00)00197-x

[2] Fujii T, Ishitani A, Geraghty DE. A soluble form of the HLA-G antigen is encoded by a messenger ribonucleic acid containing intron 4. J Immunol. 1994;153: 5516–24. 7989753

[3] Kirszenbaum M, Moreau P, Gluckman E, Dausset J, Carosella E. An alternatively spliced form of HLA-G mRNA in human trophoblasts and evidence for the presence of HLA-G transcript in adult lymphocytes. Proc Natl Acad Sci U S A. 1994;91: 4209–13. 8183892 10.1073/pnas.91.10.4209 PMC43754

[4] Hviid TV, Hylenius S, Rorbye C, Nielsen LG. HLA-G allelic variants are associated with differences in the HLA-G mRNA isoform profile and HLA-G mRNA levels. Immunogenetics. 2003;55: 63–79. 12712263 10.1007/s00251-003-0547-z

[5] Paul P, Cabestre FA, Ibrahim EC, Lefebvre S, Khalil-Daher I, Vazeux G, et al Identification of HLA-G7 as a new splice variant of the HLA-G mRNA and expression of soluble HLA-G5, -G6, and -G7 transcripts in human transfected cells. Hum Immunol. 2000;61: 1138–49. 11137219 10.1016/s0198-8859(00)00197-x

[6] Carosella ED, Gregori S, LeMaoult J. The tolerogenic interplay(s) among HLA-G, myeloid APCs, and regulatory cells. Blood. 2011;118: 6499–505. 10.1182/blood-2011-07-370742 21960588

[7] Veit TD, Vianna P, Chies JAB. HLA-G—From Fetal Tolerance to a Regulatory Molecule in Inflammatory Diseases. Curr Immunol Rev. 2010;6: 1–15.

[8] Gonzalez A, Rebmann V, LeMaoult J, Horn PA, Carosella ED, Alegre E. The immunosuppressive molecule HLA-G and its clinical implications. Crit Rev Clin Lab Sci. 2012;49: 63–84. 10.3109/10408363.2012.677947 22537084

[9] Shiroishi M, Kuroki K, Ose T, Rasubala L, Shiratori I, Arase H, et al Efficient leukocyte Ig-like receptor signaling and crystal structure of disulfide-linked HLA-G dimer. J Biol Chem. 2006;281: 10439–47. 16455647 10.1074/jbc.M512305200

[10] Apps R, Gardner L, Sharkey AM, Holmes N, Moffett A. A homodimeric complex of HLA-G on normal trophoblast cells modulates antigen-presenting cells via LILRB1. Eur J Immunol. 2007;37: 1924–37. 17549736 10.1002/eji.200737089 PMC2699429

[11] Zhong M, Weng X, Liang Z, Lu S, Li J, Chen X, et al Dimerization of soluble HLA-G by IgG-Fc fragment augments ILT2-mediated inhibition of T-cell alloresponse. Transplantation. 2009;87: 8–15. 10.1097/TP.0b013e31818b6141 19136885

[12] Baricordi OR, Stignani M, Melchiorri L, Rizzo R. HLA-G and inflammatory diseases. Inflamm Allergy Drug Targets. 2008;7: 67–74. 18691135 10.2174/187152808785107615

[13] Fainardi E, Castellazzi M, Stignani M, Morandi F, Sana G, Gonzalez R, et al Emerging topics and new perspectives on HLA-G. Cell Mol Life Sci. 2011;68: 433–51. 10.1007/s00018-010-0584-3 21080027 PMC11114687

[14] Khosrotehrani K, Le Danff C, Reynaud-Mendel B, Dubertret L, Carosella ED, Aractingi S. HLA-G expression in atopic dermatitis. J Invest Dermatol. 2001;117: 750–2. 11564188 10.1046/j.0022-202x.2001.01487.x

[15] Wiendl H, Behrens L, Maier S, Johnson MA, Weiss EH, Hohlfeld R. Muscle fibers in inflammatory myopathies and cultured myoblasts express the nonclassical major histocompatibility antigen HLA-G. Ann Neurol. 2000;48: 679–84. 11026456

[16] Aractingi S, Briand N, Le Danff C, Viguier M, Bachelez H, Michel L, et al HLA-G and NK receptor are expressed in psoriatic skin: a possible pathway for regulating infiltrating T cells? Am J Pathol. 2001;159: 71–7. 11438456 10.1016/S0002-9440(10)61675-6 PMC1850403

[17] Kapasi K, Albert SE, Yie S, Zavazava N, Librach CL. HLA-G has a concentration-dependent effect on the generation of an allo-CTL response. Immunology. 2000;101: 191–200. 11012772 10.1046/j.1365-2567.2000.00109.x PMC2327080

[18] Carosella ED, Moreau P, Aractingi S, Rouas-Freiss N. HLA-G: a shield against inflammatory aggression. Trends Immunol. 2001;22: 553–5. 11574278 10.1016/s1471-4906(01)02007-5

[19] Kanai T, Fujii T, Kozuma S, Yamashita T, Miki A, Kikuchi A, et al Soluble HLA-G influences the release of cytokines from allogeneic peripheral blood mononuclear cells in culture. Mol Hum Reprod. 2001;7: 195–200. 11160846 10.1093/molehr/7.2.195

[20] Torres MI, Le Discorde M, Lorite P, Rios A, Gassull MA, Gil A, et al Expression of HLA-G in inflammatory bowel disease provides a potential way to distinguish between ulcerative colitis and Crohn's disease. Int Immunol. 2004;16: 579–83. 15039388 10.1093/intimm/dxh061

[21] Mitsdoerffer M, Schreiner B, Kieseier BC, Neuhaus O, Dichgans J, Hartung HP, et al Monocyte-derived HLA-G acts as a strong inhibitor of autologous CD4 T cell activation and is upregulated by interferon-beta in vitro and in vivo: rationale for the therapy of multiple sclerosis. J Neuroimmunol. 2005;159: 155–64. 15652415 10.1016/j.jneuroim.2004.09.016

[22] Verbruggen LA, Rebmann V, Demanet C, De Cock S, Grosse-Wilde H. Soluble HLA-G in rheumatoid arthritis. Hum Immunol. 2006;67: 561–7. 16916651 10.1016/j.humimm.2006.03.023

[23] Rizzo R, Farina I, Bortolotti D, Galuppi E, Rotola A, Melchiorri L, et al HLA-G may predict the disease course in patients with early rheumatoid arthritis. Hum Immunol. 2013. 10.1016/j.humimm.2012.11.024 23228398

[24] Rizzo R, Rubini M, Govoni M, Padovan M, Melchiorri L, Stignani M, et al HLA-G 14-bp polymorphism regulates the methotrexate response in rheumatoid arthritis. Pharmacogenet Genomics. 2006;16: 615–23. 16906016 10.1097/01.fpc.0000230115.41828.3a

[25] Ferraz MB, Oliveira LM, Araujo PM, Atra E, Tugwell P. Crosscultural reliability of the physical ability dimension of the health assessment questionnaire. J Rheumatol. 1990;17: 813–7. 2388204

[26] Schutt P, Schutt B, Switala M, Bauer S, Stamatis G, Opalka B, et al Prognostic relevance of soluble human leukocyte antigen-G and total human leukocyte antigen class I molecules in lung cancer patients. Hum Immunol. 2010;71: 489–95. 10.1016/j.humimm.2010.02.015 20156510

[27] Rebmann V, Lemaoult J, Rouas-Freiss N, Carosella ED, Grosse-Wilde H. Report of the Wet Workshop for Quantification of Soluble HLA-G in Essen, 2004. Hum Immunol. 2005;66: 853–63. 16216668 10.1016/j.humimm.2005.05.003

[28] Verloes A, Van de Velde H, LeMaoult J, Mateizel I, Cauffman G, Horn PA, et al HLA-G expression in human embryonic stem cells and preimplantation embryos. J Immunol. 2011;186: 2663–71. 10.4049/jimmunol.1001081 21248264

[29] Rebmann V, Switala M, Eue I, Schwahn E, Merzenich M, Grosse-Wilde H. Rapid evaluation of soluble HLA-G levels in supernatants of in vitro fertilized embryos. Hum Immunol. 2007;68: 251–8. 17400060 10.1016/j.humimm.2006.11.003

[30] Lahiri DK, Nurnberger JI Jr. A rapid non-enzymatic method for the preparation of HMW DNA from blood for RFLP studies. Nucleic Acids Res. 1991;19: 5444 1681511 10.1093/nar/19.19.5444 PMC328920

[31] Hviid TV, Hylenius S, Hoegh aM, Kruse C, Christiansen OB. HLA-G polymorphisms in couples with recurrent spontaneous abortions. Tissue Antigens. 2002;60: 122–32. 12392506 10.1034/j.1399-0039.2002.600202.x

[32] Cordero EAA, Veit TD, Silva MAL, Jacques SMC, Silla LMDR, Chies JAB. HLA-G polymorphism influences the susceptibility to HCV infection in sickle cell disease patients. Tissue Antigens. 2009: 308–13. 10.1111/j.1399-0039.2009.01331.x 19775370

[33] Rizzo R, Rubini M, Govoni M, Padovan M, Melchiorri L, Stignani M, et al HLA-G 14-bp polymorphism regulates the methotrexate response in rheumatoid arthritis. Pharmacogenetics and genomics. 2006;16: 615–23. 16906016 10.1097/01.fpc.0000230115.41828.3a

[34] Fainardi E, Rizzo R, Melchiorri L, Vaghi L, Castellazzi M, Marzola A, et al Presence of detectable levels of soluble HLA-G molecules in CSF of relapsing-remitting multiple sclerosis: relationship with CSF soluble HLA-I and IL-10 concentrations and MRI findings. J Neuroimmunol. 2003;142: 149–58. 14512174 10.1016/s0165-5728(03)00266-2

[35] Prigione I, Penco F, Martini A, Gattorno M, Pistoia V, Morandi F. HLA-G and HLA-E in patients with juvenile idiopathic arthritis. Rheumatology (Oxford). 2011;50: 966–72. 10.1093/rheumatology/keq418 21186170

[36] Gonzalez A, Alegre E, Arroyo A, LeMaoult J, Echeveste JI. Identification of circulating nonclassic human leukocyte antigen G (HLA-G)-like molecules in exudates. Clin Chem. 2011;57: 1013–22. 10.1373/clinchem.2010.159673 21527645

[37] Apps R, Sharkey A, Gardner L, Male V, Kennedy P, Masters L, et al Ex vivo functional responses to HLA-G differ between blood and decidual NK cells. Molecular human reproduction. 2011;17: 577–86. 10.1093/molehr/gar022 21471023 PMC3160205

[38] Zilberman S, Schenowitz C, Agaugue S, Benoit F, Riteau B, Rouzier R, et al HLA-G1 and HLA-G5 active dimers are present in malignant cells and effusions: the influence of the tumor microenvironment. European journal of immunology. 2012;42: 1599–608. 10.1002/eji.201141761 22678912

[39] Kuroki K, Hirose K, Okabe Y, Fukunaga Y, Takahashi A, Shiroishi M, et al The long-term immunosuppressive effects of disulfide-linked HLA-G dimer in mice with collagen-induced arthritis. Hum Immunol. 2013;74: 433–8. 10.1016/j.humimm.2012.11.060 23276819

[40] Naji A, Menier C, Maki G, Carosella ED, Rouas-Freiss N. Neoplastic B-cell growth is impaired by HLA-G/ILT2 interaction. Leukemia. 2012;26: 1889–92. 10.1038/leu.2012.62 22441169

[41] Rosado S, Perez-Chacon G, Mellor-Pita S, Sanchez-Vegazo I, Bellas-Menendez C, Citores MJ, et al Expression of human leukocyte antigen-G in systemic lupus erythematosus. Hum Immunol. 2008;69: 9–15. 10.1016/j.humimm.2007.11.001 18295670

[42] Rizzo R, Hviid TV, Govoni M, Padovan M, Rubini M, Melchiorri L, et al HLA-G genotype and HLA-G expression in systemic lupus erythematosus: HLA-G as a putative susceptibility gene in systemic lupus erythematosus. Tissue Antigens. 2008;71: 520–9. 10.1111/j.1399-0039.2008.01037.x 18380776

[43] Rebmann V, van der Ven K, Pässler M, Pfeiffer K, Krebs D, Grosse-Wilde H. Association of soluble HLA-G plasma levels with HLA-G alleles. Tissue antigens. 2001;57: 15–21. 11169254 10.1034/j.1399-0039.2001.057001015.x

[44] Chen X-Y, Yan W-H, Lin a, Xu H-H, Zhang J-G, Wang X-X. The 14 bp deletion polymorphisms in HLA-G gene play an important role in the expression of soluble HLA-G in plasma. Tissue antigens. 2008;72: 335–41. 10.1111/j.1399-0039.2008.01107.x 18700878

