# Supplementary material for: The Paradox of High Availability and Low Recognition of Soluble HLA-G by LILRB1 Receptor in Rheumatoid Arthritis Patients
Source: PLoS One. 2015 Apr 8;10(4):e0123838. doi: 10.1371/journal.pone.0123838 (PMC4390237; doi:10.1371/journal.pone.0123838)
Supplement: S1 File — Table A, Correlations of RA disease parameters and levels of sHLA-G molecules with their LILRB1 recognition. Table B, Relationship of RA treatment and levels of sHLA-G molecules with their LILRB1 recognition. (DOCX) [file pone.0123838.s001.docx]

**Table A. Correlations of RA disease parameters and levels of sHLA-G molecules with their LILRB1 recognition.**

|  |  | **sHLA-G** | | **LILRB1 recognition** | |
| --- | --- | --- | --- | --- | --- |
| **Clinical parameter** | **n** | **r_Spearman_** | **P-value** | **r_Spearman_** | **P-value** |
| **ESR** | 60 | 0.244 | 0.060 | 0.099 | 0.450 |
| **CRP** | 61 | 0.144 | 0.270 | 0.111 | 0.395 |
| **Swollen joints** | 63 | 0.049 | 0.706 | 0.170 | 0.182 |
| **Tender joints** | 61 | -0.080 | 0.539 | 0.158 | 0.222 |
| **VAS (patient)** | 56 | -0.093 | 0.496 | 0.101 | 0.457 |
| **VAS (evaluator)** | 56 | 0.105 | 0.442 | 0.061 | 0.657 |
| **DAS28-ESR** | 57 | 0.093 | 0.489 | 0.190 | 0.156 |
| **DAS28-CRP** | 56 | 0.003 | 0.980 | 0.157 | 0.248 |
| **CDAI** | 56 | 0.057 | 0.676 | 0.210 | 0.121 |

ESR = erythrocyte sedimentation rate; CRP = C-reactive protein; VAS = visual analogue scale; DAS = disease activity score; CDAI clinical disease activity index

**Table B. Relationship of RA treatment and levels of sHLA-G molecules with their LILRB1 recognition.**

|  |  |  | **sHLA-G** | | **LILRB1 recognition** | |
| --- | --- | --- | --- | --- | --- | --- |
| **Treatment** |  | **n** | **Mean rank** | **P-value^a^** | **Mean rank** | **P-value^a^** |
| **Methotrexate** | Yes | 58 | 33.2 | 0.200 | 32.4 | 0.031 |
|  | No | 10 | 41.9 |  | 46.4 |  |
| **Prednisone** | Yes | 39 | 35.8 | 0.527 | 35.4 | 0.669 |
|  | No | 29 | 32.7 |  | 33.4 |  |
| **Leflunomide** | Yes | 14 | 35.9 | 0.773 | 32.5 | 0.651 |
|  | No | 54 | 34.2 |  | 35.0 |  |
| **Chloroquine** | Yes | 5 | 34.8 | 0.982 | 38.6 | 0.648 |
|  | No | 63 | 34.5 |  | 34.2 |  |
| **Sulphasalazine** | Yes | 3 | 32.3 | 0.898 | 37.2 | 0.786 |
|  | No | 65 | 34.1 |  | 33.8 |  |
| **Infliximab** | Yes | 3 | 38.3 | 0.757 | 45.5 | 0.341 |
|  | No | 65 | 34.3 |  | 34.0 |  |

^a^Mann-Whitney test
